# Emerging roles of sortilin in affecting the metabolism of glucose and lipid profiles

**DOI:** 10.17305/bjbms.2021.6601

**Published:** 2021-11-15

**Authors:** Xin Su, Linjian Chen, Xiang Chen, Cuilian Dai, Bin Wang

**Affiliations:** Department of Cardiology, the Xiamen Cardiovascular Hospital of Xiamen University, Xiamen, Fujian, China

**Keywords:** Sortilin, dyslipidemia, insulin resistance, diabetes mellitus, cardiovascular disease

## Abstract

Dyslipidemia has recently been identified as an important factor in modulating the progression of several health conditions, grouped as cardiometabolic syndrome and including obesity, insulin resistance, and atherosclerosis. Among multiple factors which regulate the development of cardiometabolic syndrome, sortilin has been found in multiple cell types, such as adipocyte, hepatocyte, and macrophage, suggesting that sortilin is correlated to the development and the severity of cardiometabolic syndrome. Consistently, several genome-wide association studies and basic experimental research studies are being conducted to find novel gene loci involved in regulating the pathological progression of cardiometabolic syndrome. According to these data, both sortilin 1 gene and sortilin protein have an important function in regulating the circulating lipid and glucose metabolism resulting in modulation of disease progression. In this comprehensive review, we summarize the recent research results regarding sortilin function in modulating the circulating lipid and glucose metabolism. Moreover, we also discuss and analyze the emerging evidence elucidating the potential mechanisms by which sortilin affect synthesis and secretion of lipid and glucose.

## INTRODUCTION

According to the eye-catching reports worldwide, dyslipidemia has been demonstrated as one of the most essential factors which is strongly associated with the pathological development of several health problems, such as hypertension, overweight/obesity, diabetes mellitus, and atherosclerotic related cardiovascular diseases, which are currently given a conception as cardiometabolic syndrome [1]. Actually, due to the results of epidemiological studies, dyslipidemia has become one of the most pressing issues worldwide during the past several decades, posing serious threat to human health and promoting a high mortality [2]. In addition, it is also worth noting that dyslipidemia, characterized by elevated serum concentrations of low-density lipoprotein cholesterol (LDL-C), very LDL-C (VLDL-C), and decreased serum concentrations of high-density lipoprotein cholesterol, has also been considered as the most vital modifiable factor hand in hand with the development and the severity of cardiometabolic syndrome [3]. In details, it has been demonstrated that the progression of LDL particle intruding into sub-endothelium within circulation induced by the macrophage is intimately involved in the pathogenesis of atherosclerotic related cardiovascular diseases; by contrast, the lower serum concentrations of LDL-C induced using lipid-lowering agents, such as statins or Ezetimibe, are closely associated with the inhibition of atherosclerotic lesions which further reduced the risk and suppressed the severity of cardiometabolic syndromes [4].

As is known to us, several metabolic related cell types, such as the adipocyte, the macrophage, and the hepatocyte, have been confirmed as the crucial cells participating in circulating lipid metabolism. On the other hand, increasing evidence has put forward that several lipid metabolic related genes, such as low density lipoprotein receptor (*LDLR*) gene and proprotein convertase subtilisin/kexin type 9 (*PCSK9*) gene, embrace a vital function in regulating circulating and intracellular levels of lipid profiles [5-7]. Noteworthy, multiple genetic studies revealed that the progression of atherosclerosis was strongly heritable, since the variations characterized by significantly elevated serum concentrations LDL-C in those genes. Further understanding of the correlation between the mutations of lipid metabolic related genes with the pathology of atherosclerosis could put forward an efficient treatment strategy to inhibit the process of cardiometabolic disorders [8]. Although the investigations of rare Mendelian disorders with the serum concentrations LDL-C provide a novel insight of the modulatory genetics on lipid metabolism [9], the disorders could not explain the various risks of dyslipidemia and its related metabolic diseases in general populations, suggesting that there might be other undiscovered gene which holds essential role in the variability of level of lipid profiles.

During the recent past decades, the genome wide association studies (GWAS) have given multiple new gene loci which are involved in circulating lipid catabolism. Notably, among these gene loci, the chromosome 1p13 has recently been demonstrated to embrace an important role in modulating the metabolism of lipid and glucose. According to the reports, this loci harbored multiple metabolic related genes, including cadherin EGF LAG seven-pass G-type receptor 2 gene, proline/serine-rich-coiled protein 1 gene, and sortilin 1 (*SORT1*) gene [10]. Among those three genes, the single gene polymorphisms single nucleotide polymorphisms (SNP) of *SORT1* gene in linking dyslipidemia and the pathogenesis of cardiometabolic syndrome has been published by several large-scale studies [11,12]. Besides, as a family member of a larger vacuolar protein sorting 10 protein (Vps10p) family, sortilin embraces the capability in binding to diverse ligands within different cells [13]. In the present comprehensive review, the understanding of sortilin in regulating circulating or intracellular lipid metabolism has been summarized. In addition, the mechanisms whereby sortilin regulates the metabolic progression of lipid and glucose in adipocyte, hepatocyte, and macrophage are also proposed for elucidating the biological function of sortilin.

## BASIC FEATURES OF *SORT1* GENE AND SORTILIN PROTEIN

As mentioned above, sortilin belongs to the protein family of Vps10p receptors. Emerging evidence provided by computational and functional proteomics research has demonstrated that there is a 700 amino-acid in the N-terminus of domain of Vps10p (Vps10p-D), which could further combine to ligands site and mediate the intracellular transportation of synthesized protein between trans-Golgi network (TGN) and endosomes [14,15] Thereby, Vps10p has been identified as a novel type-1 trans-membrane protein that is mainly found on the membrane [16]. Aside from sortilin, the Vps10p family protein also contains four other members, as sorting protein-related receptor with A-type repeats and sortilin-related receptor central nervous system (CNS) expressed 1-3 [17]. Notably, sortilin is considered as the first discovered non-G-protein-coupled receptor in mammals.

On the other hand, results from the protein crystallization research revealed that the precursor of sortilin contained a relatively long N-terminal peptide and a relatively short C-terminal cytoplasmic tail which could combine with the receptor on the surface of Golgi [18]. The mature sortilin, as a 95 kDa peptide chain with 874 amino acids, is majorly localized on the TGN. Nevertheless, a minor fraction of mature sortilin could be found on the membrane. After combining to a ligand, the mature sortilin could form a b-propeller structure that contains several binding sites [19].

Sortilin has firstly been found to be abundant within the CNS and been described as a neurotransmitter receptor which regulates neuronal cells survival and death; however, it has recently been found in multiple cell types, such as adipocyte, hepatocyte, and macrophage [20]. At cellular level, sortilin acts as a linking protein between the TGN, endosome, lysosome, and induces multiple lipoprotein including lipoprotein lipase (LPL), apolipoprotein E (Apo-E), and Apo-B100 [21]. Otherwise, at membrane level, sortilin acts as an uptake receptor which mediates the endocytosis progression of native LDL by the macrophage [22]. Furthermore, the trafficking properties of sortilin were demonstrated to take part in protein cargo sorting [23]. Concerning this notion, several ligands of sortilin, such as Apo-B100, could be transported from endosome to Golgi apparatus. Other ligands, such as Apo-E, are targeted on lysosomal degradation [24]. Conclusively, the findings demonstrated above indicate that sortilin is importantly correlated to the pathological development and the severity of cardiometabolic syndrome.

## ROLE OF SORTILIN IN MODULATING LIPID METABOLISM

Since it has been proposed that sortilin is correlated with cardio-metabolic disorder diseases, the current focus is transferring to elucidating the important effect of sortilin as well as exploring mechanisms by which *SORT1* gene or sortilin protein affects the risk of dyslipidemia [25]. Emerging evidence provided by diverse research suggests that sortilin has been identified as a new modulator of intracellular or serum lipid metabolism. The summary of sortilin in modulating lipid metabolism was listed in Table 1.

**TABLE 1:**
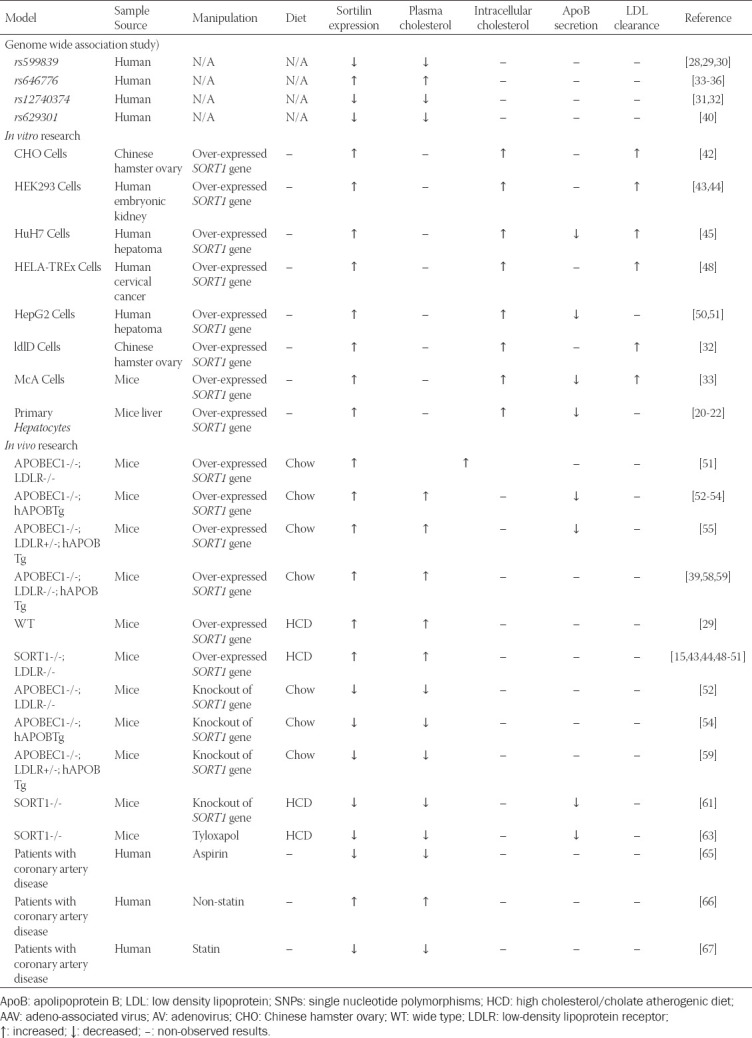
Summary of sortilin in modulating lipid metabolism

## *SORT1* SNPs IN LIPID METABOLISM

At present, extensive sequencing analysis of human *SORT1* gene interval is being conducted to identify SNPs of *SORT1* gene by GWAS. As shown, a close relationship between *SORT1* gene SNPs with circulating levels of serum lipid profiles has been put forward by different research from diverse countries. For instance, the first research, which found the relationship between *SORT1* gene SNPs and the risk of dyslipidemia, used data of approximately 9,000 patients and discovered that SNPs of 18 gene loci had relationship with alterations of serum lipid profiles. Among these loci, the 1p13 locus was shown to be correlated with the *SORT1* gene, and a proxy for 1p13 locus SNP was afterward confirmed to influence the development of atherosclerotic cardiovascular diseases [11]. Likewise, Schadt et al. also identified *SORT1* gene SNP had a close relationship with the altered serum LDL-C [26]. Results from GWAS of the Third United States National Health and Nutrition Examination Survey also provided the similar relationship, demonstrating that the *SORT1* gene SNP play embraces a significant function in modulating serum lipid profile [27].

Due to the basic experimental advances, multiple eye-catching breakthroughs have been made to explain several novel *SORT1* gene SNPs, such as *rs646767* SNP, *rs12734074* SNP, and *rs623901* SNP, and serum lipid and glucose levels. Recently, with in-depth investigations, Nakayama et al. enrolled more than 21,000 Japanese subjects and demonstrated that the *rs599839* SNP was correlated with significantly decreased circulating concentrations of atherogenic related lipid profiles, including TG and LDL-C [28]. In addition, these results have also been replicated in diverse investigations from several European countries, indicating that the G-allele within *rs599839* SNP was positively associated with the risk and the development of atherosclerotic cardiovascular diseases [29,30].

On the other hand, a study revealed that *rs12740374* SNP was strongly correlated with circulating levels of LDL-C [31]. Similar with this finding, Musunuru et al. also put forward that the *rs12740374* SNP promoted the expression levels of CCAAT/enhancer binding protein (C/EBP) within hepatocyte, inducing to the lower concentrations of LDL-C and tge suppressed production of VLDL particles [32]. Besides, this result also suggests that *SORT1* gene SNP modulates the risk of dyslipidemia potentially through, at least partly, regulating the gene expression content of *C/EBP*.

Recently, the *rs646776* SNP was also identified to be correlated with elevated circulating concentrations of LDL-C in children and youth [33]. Aside from the modulatory role in serum LDL-C, the *rs646776* SNP also presented a genotype-specific discordance in serum LDL-C concentrations which was greater within younger participants compared to those within the older participants [34]. Consistent relationship was further found in numerous research which focuses on participants from diverse countries [35-38]. By summarizing the results of these researches, Zeng et al. focused on the correlation between *rs646776* SNP with circulating lipid levels in the Chinese Han population. Interestingly, the authors confirmed that the *rs646776* SNP could up-regulate the *SORT1* gene expression and as a consequence, increase the circulating LDL-C levels [39]. More recently, another study enrolled a cohort of 2,800 African-Americans and observed that a novel *SORT1* gene SNP, named *rs629301* SNP, was associated with the plasma concentrations of LDL-C and VLDL-C [40]. Taken together, these results indicate that several *SORT1* gene SNPs hold a genetic link between serum lipid hemostasis with the pathogenesis of cardiometabolic syndrome, including obesity, hypertension, and the atherosclerotic related coronary artery disease.

## SORTILIN IN LIPID METABOLISM *IN VITRO*

According to the findings from several *in vitro* researches, a direct relationship between sortilin and lipid metabolism is well-established using diverse cell types [41-43]. For instance, Patel et al. used the macrophages that were derived from the bone marrow of *SORT1*-deficient mice and found that these macrophages had significantly reduced uptake rate of LDL-C which subsequently led to an inhibition of foam cells formation. On the contrary, over-expressed *SORT1* gene within macrophage induced up-regulated LDL uptake, indicating that increased *SORT1* gene expression within macrophage could lead to increased intercellular lipid storage [44].

Besides the important function of sortilin in macrophage, other research used HEK293 cells and found that sortilin led to increased total cholesterol levels within circulation. Kjolby et al. and Gustafsen et al. found that sortilin could co-localize with *PCSK9* in the TNG, thus enhance the secretion of *PCSK9* from HEK293 cells, inducing a degradation of LDL receptor and a reduced clearance rate of LDL-C [45,46]. Further, study by Linsel-Nitschke et al. also revealed that over-expression of *SORT1* gene resulted in increased endocytosis of LDL-C rate within HEK293 cells [47].

Eye-catchingly, a recent research demonstrated that the primary hepatocyte isolated from *SORT1* gene-knockout mice exhibited significantly reduced LDL-C contents compared with those within wide-type (WT) mice. With in-depth research, the authors found the expression levels of lipogenic related genes, including fatty acid binding protein-4 (FABP-4) gene and stearoyl-CoA desaturase-1 gene, were down-regulated remarkably within the hepatocytes isolated from *SORT1* gene-knockout mice, proposing a potential pathway by which sortilin influences the intracellular lipid concentration within the hepatocyte [48].

The important function of sortilin in modulating intracellular lipid metabolism in other cell types has also begun to gain appreciation. Noteworthy, Strong et al. provided evidence that over-expression of *SORT1* gene in diverse cell lines could induce an increase in LDL clearance [15,42]. In addition, Tveten transfected plasmids, which contained WT *SORT1* gene or mutant *SORT1* gene, in HeLa TREx cells and demonstrated a positive relationship between the contents of sortilin on the cell surface and the concentrations of LDL bound, revealing that sortilin could not elevate the binding of LDL through an intracellular potential mechanism whereas on the cell surface [49]. In conclusion, these results revealed a functional role linking expression of *SORT1* gene and sortilin protein with intracellular lipid levels.

## SORTILIN IN LIPID METABOLISM *IN VIVO*

It is worth noting that several reports cumulatively revealed that knockout of *SORT1* gene reduced the serum lipid levels and the atherosclerotic plaque area significantly. Patel et al. observed a significant reduction of atherosclerosis in *LDLR* (-/-) mice with transplanted *SORT1*(-/-) bone marrow, without an effect on the serum concentrations LDL-C [44]. Furthermore, lack of *SORT1* expression leads to a robust drop of LDL-C and total cholesterol within circulation, however, the hepatocyte-specific over-expression of *SORT1* gene induced the significantly increased concentrations of LDL-C and TG [44]. Consistent results were presented in several basic experimental studies, conducted by Rabinowich et al. and Kjolby et al., which showed decreased circulating levels of intermediate density lipoprotein in *SORT1*-knockout mice and in *SORT1/LDLR*-double knockout (DKO) mice [12,45,48]. In addition, the mice with *LDLR*-deficiency and over-expressed the *SORT1* gene also displayed increased sortilin protein expression and accelerated circulating LDL-C clearance. Simultaneously, when *SORT1*(-/-) mice were treated intravenously with tyloxapol, a special inhibitor of LPL, the VLDL particles were smaller than that in WT mice [12]. Using *SORT1*(-/-) mice on a chow diet, Strong et al. found that *SORT1* deficiency had a close relationship with reduced VLDL production and increased LDL catabolism [15,42]. Consistently, Mortensen et al. reported that *SORT1/APOE*-DKO mice displayed slightly lower cholesterol levels than that in *APOE*(-/-) mice with *SORT1* over-expression [50]. More Recently, Hagita et al. showed that in the *LDLR*(-/-) mice model, *SORT1* deficiency could lead to a reduction in body weight and in plasma TG levels, indicating that *SORT1* could influence the development of obesity [51].

Importantly, several studies concerned the relationship between the levels of sortilin and lipid in clinical patients. Ogawa et al. reported that in aspirin-treated CAD patients, the platelet aggregation could induce solute sortilin release, which could be suppressed by aspirin. In addition, plasma solute sortilin was higher in patients with hypertension, dyslipidemia and diabetes without coronary artery disease than in patients with coronary artery disease who underwent the aspirin therapy. In these patients, the levels of circulating solute sortilin hold positive relationship with circulating levels of LDL-C and TG [52]. In 2017, Hu et al. demonstrated that serum levels of *PCSK9* and sortilin were significantly up-regulated in patients with coronary artery disease. Furthermore, the *PCSK9* was independent related to the expression levels of sortilin, indicating that sortilin could mediate the secretion of *PCSK9* in plasma [53]. As firmly reported, *PCSK9* induces reduced LDL-C clearance within circulation through the degradation of LDLR, we could speculate that sortilin may facilitate the risk of atherosclerotic related diseases through the expression of *PCSK9*.

## MECHANISMS OF SORTILIN IN INTRACELLULAR LIPID METABOLISM

### Hepatocyte

As described previously, hepatocytes produce and release multiple proteins which embrace an eye-catching role in regulating lipid metabolism. Thus, hepatocyte is considered as a key factor in the risk of dyslipidemia [54]. Under the condition of hepatic related diseases, such as the non-alcoholic fatty liver diseases, the metabolic processes of serum lipid profiles are impaired owing to the aberrant lipid metabolism by hepatocyte [55,56]. In addition, under that pathological condition, excessive circulating lipid profiles may enter into hepatocytes, which subsequently cause excessive intracellular lipid load [57]. However, the accurate mechanisms whereby sortilin influences the intracellular lipid catabolism in hepatocytes need to be further elucidated.

Actually, several underlying mechanisms have been provided to further explain the association between sortilin with the risk and the development of dyslipidemia. First, as described, LDL-C is produced by VLDL through lipolysis. Therefore, the major mechanism is that *SORT1* gene in hepatocyte may promote VLDL lipolysis by interacting with ApoB-100 and as a consequence, increases circulating LDL-C level. These findings indicate that sortilin combines Apo-B100 within Golgi apparatus and resultantly regulates the formation of VLDL within hepatocyte and LDL within circulation [43]. Second, sortilin has been found to interact with *PCSK9*; additionally, sortilin promotes *PCSK9* production and secretion in hepatocytes. Notably, the up-regulated *PCSK9* could bind to LDLR and trigger the degradation process of LDLR, which could induce a significant reduction of LDL-C clearance within circulation [46,58]. Third, it is also worth paid attention that the mice with *SORT1*-deficiency also present a decreased released rate of VLDL, accompanied with the reduction of circulating concentrations of LDL-C. Recently, an independent research demonstrated that the hepatocyte isolated from the mice with *SORT1*-deficiency presented the decreased risk of diet-induced hepatic steatosis. After intervening mice with AF38469, as an orally bioactive inhibitor of sortilin, the authors demonstrated that these mice displayed reduced circulating concentrations of LDL-C and relatively lower expression levels of pro-inflammatory cytokines within hepatocytes. Synchronously, AF38469 intervention has also been reported to be correlated with lower hepatic VLDL released and higher expression levels of cholesterol 7a-hydrolase [59].

Nonetheless, increasing results demonstrated that over-expression of *SORT1* lowered circulating lipid levels. One of the most interesting researches conducted by Musunuru et al. revealed that the hepatocyte with over-expression of *SORT1* gene presented lower VLDL secretion rate through transporting VLDL to the lysosome, leading to reduced serum levels of cholesterol. Consequently, this result indicates a negative modulatory effect of sortilin in controlling intracellular lipid metabolism. Moreover, the authors also found that over-expression of *SORT1* gene in hepatocyte could induce the decreased Apo-B100 synthesis, putting forward a now pathway of sortilin in suppressing VLDL production and release through, at least partly, binding to Apo-B100 within Golgi apparatus [32].

**FIGURE 1:**
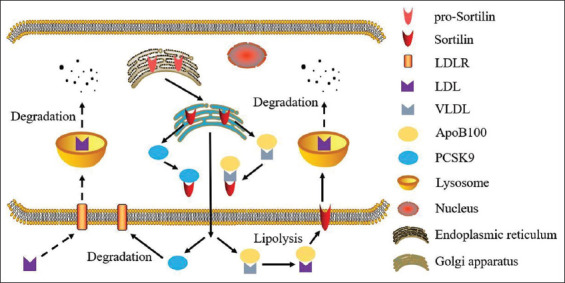
Mechanisms whereby sortilin influences lipid metabolism in hepatocytes. Sortilin could promote VLDL lipolysis by interacting with apolipoprotein B-100 and resultantly increasing circulating levels of LDL-C. LDLR: low-density lipoprotein receptor; Apo-B100: apolipoprotein B100; LDL: low-density lipoprotein; VLDL: very low-density lipoprotein; PCSK9: proprotein convertase subtilisin/kexin type 9.

More recently, an independent study suggested an interesting function for sortilin as an alternative LDLR in the liver. According to this suggestion, we could speculate that sortilin may increase the LDL endocytosis into hepatocytes and enhance the clearance of lipoprotein from circulation [47,60]. Conclusively, these potential mechanisms could propose that sortilin modulates both serum and intracellular lipid levels within hepatocytes. The mechanism is shown in Figure 1.

The discordant results mentioned above provide contradictory functions of sortilin on serum levels of VLDL-C which are probably induced by the diverse experimental mouse models. Otherwise, it still remains to be further explored whether the alteration of *SORT1* gene expression results in dyslipidemia and its related metabolic diseases.

### Adipocyte

Overweight and obesity have been shown to be strongly associated with multiple chronic diseases, including dyslipidemia, hypertension, and type 2 diabetes mellitus [61,62]. At present, obesity is being identified as one of the most vital risk factors of cardio-metabolic syndrome, which resultantly induces the most common cause of death [63]. With in-depth research, it is being elucidated that one of the hallmarks of obesity is excessive storage of TG within the adipocyte that is the predominant cell type of adipose tissue. At present, the adipocyte is considered to be not only a huge repository of fat but also an essential source of adipocytokines [64,65]. Under obese condition, the adipocyte is hypertrophic and is involved in regulating serum lipid metabolism. Consistent with this notion, increasing results revealed that the adipose tissue contained such abundant adipose-derived mesenchymal stem cells (AMSCs) which could differentiate into mature adipocytes [66]. As is known to us, the differentiation process of AMSCs is separated into about three stage, as the early, the intermediate, and the terminal stage, hand in hand with a complex transcription factors, such as C/EBPα/β and proliferator activated receptor γ. To be more specific, C/EBPβ embraces an essential function during adipocyte differentiation at the early stage [67], which subsequently stimulates the expression of C/EBPα within the intermediate stage [68]. Notably, the up-regulated C/EBPα could further lead to the transcription of numerous adipogenic differentiation related biomarkers, such as FABP-4 and fatty acid synthetase, and could maintain mature phenotype of adipocyte in the terminal stage [69]. Thereby, the aberrant adipogenic differentiation of AMSCs induces hyperplasia and hypertrophy of adipocytes, resultantly facilitating the pathological process of cardiometabolic disorder diseases.

The important functions of sortilin in modulating lipid catabolism within an adipocyte are gaining substantial appreciation since the adipocyte could produce and release multiple lipid metabolic-related proteins. The first results which showed an association between sortilin with the adipogenic differentiation of pre-adipocytes was reported by Breitling et al., who confirmed that expression content of *SORT1* gene was increased significantly with the progression of adipogenesis. Some SNPs of *SORT1* gene has been well-demonstrated to elucidate the functions of sortilin in controlling adipocytic lipid catabolism. For instance, the SNP of *rs599839*, which was shown previously to affect serum concentrations of LDL-C, played an important role in controlling adipogenesis [70]. Consistent with these findings, another study used SV129/BL6 mice and found that sortilin down-regulated the TG concentration within 3T3-L1 preadipocytes and suppressed the adipogenic differentiation via binding to a vital surface receptor, named the adipogenic limiting receptor δ-like protein 1 (DLK1) [71]. According to the previous reports, DLK1 has been found to be only expressed on the surface of pre-adipocytes and is being considered as a pre-adipocyte marker [72,73]. It has been well-established through *in vitro* and *in vivo* investigations that altered contents of DLK1 influence the process of adipogenesis [74]. Eye-catchingly, increasing evidence has uncovered that DLK1 could be activated as an active soluble type via cleavage process induced by tumor necrosis factor α-converting enzyme (TACE). The active type of DLK1 was further verified to be a biomarker for adipocytic progenitors which suppress adipogenic differentiation [75]. Silencing DLK1 expression within 3T3-L1 pre-adipocyte mediates increased expression of C/EBPβ and C/EBPα, however, the combination of sortilin and DLK1 inhibit the degradation of DLK1 which consequently suppresses the adipogenic differentiation [76,77]. It is shown that sortilin inhibits the adipogenic progression of 3T3-L1 preadipocytes, presenting lower intracellular levels of C/EBPβ, C/EBPα, and TG contents. In conclusion, the results indicate that sortilin embraces the property of inhibiting adipogenic differentiation of pre-adipocyte [71].

Furthermore, results of another research provide a new function of sortilin in lipid homeostasis within adipocytes, which may make sortilin a therapeutic target for cardiometabolic disorders. In details, the authors used the *LDLR/SORT1*-DKO mice and confirmed that knockout of *SORT1* gene inhibited the mRNA expression of Niemann-Pick type C1-Like 1 (*NPC1L1*) gene and further reduced the mass of white adipose tissue; synchronously, knockout of *SORT1* gene brought improved function of brown adipose tissue through down-regulated expression of Kruppel-like factor 4 (*KLF4*) gene and liver X receptor (*LXR*) gene. In addition, those mice that were fed with a high cholesterol diet (HCD) presented increasing circulating levels of adiponectin [51]. Given that adiponectin is one of the most important adipokine which is negatively associated with the process of adipogenic differentiation [78,79], we could make a reasonable speculation that sortilin affect adipogenic differentiation via, at least partly, influencing adiponectin and subsequently inhibiting the development of cardiometabolic diseases through modulating the expression of *NPC1L1* gene, *KLF4* gene, and *LXR* gene.

**FIGURE 2:**
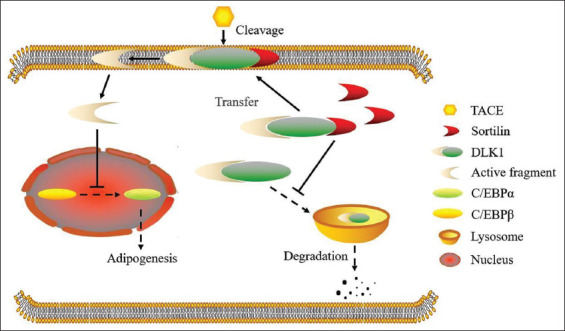
Mechanisms whereby sortilin influences lipid metabolism in adipocytes. DLK1 could be stimulated through a cleavage process induced by TACE. Sortilin inhibits the adipogenic progression of pre-adipocytes via modulating the cleavage process. TACE: TNF-α-converting enzyme; DLK1: adipogenesis-limiting receptor δ-like protein 1; C/EBP: CCAAT/enhancer binding protein.

At present, obesity and diabetes mellitus has been shown to be synergistically correlated with impaired glucose metabolism within adipocytes, which is induced by the decreased intracellular expression levels of glucose transporter 4 (GLUT4) [80-82]. Indeed, within the 3T3-L1 preadipocyte, *SORT1* gene is a component of GLUT4 vesicles [83,84]. Otherwise, another independent experimental investigation demonstrated that administration of phosphatidylinositol 3-kinase (PI3K) inhibitors could reduce expression levels of sortilin protein but not *SORT1* gene within adipocytes isolated from the chow-fed mice. The serum-starvation or suppression of the PI3K/AKT signaling pathway also reduced sortilin protein without altering *SORT1* gene expression level within 3T3-L1 preadipocyte, suggesting that the PI3K/AKT signaling pathway may induce dynamically reduced levels of sortilin and affect the intracellular lipid catabolism through DLK1 [85]. Two other independent researches indicated that, under obese condition, mice with *SORT1* gene deficiency present reduced serum TG content and increased uptake of glucose. This metabolic phenotype of adipocytes was further confirmed to be induced by decreased acid sphingomyelinase activity [86,87]. The comprehensive intracellular function of sortilin in lipid metabolism within adipocytes is shown in Figure 2.

### Macrophage

Macrophages, as one of an important type of white blood cells, have been demonstrated to engulf intracellular debris, cancer cells, and anything that has not proteins specific to healthy cells [88]. Aside from the function in modulating systemic inflammatory response, it is also well-demonstrated that macrophage also holds an important function in maintaining the circulating cholesterol homeostasis [89]. Under the condition of dyslipidemia, macrophage could ingest LDL-C and subsequently form the foam cells by special surface receptors, such as LDL receptor, cluster of differentiation 36, and scavenger receptor A. After this vital step, the foam cell is intruded into the sub-endothelial zone and facilitates the atherosclerotic lesions [90]. Besides, macrophage has also been verified to remove excessive intracellular LDL-C through a classical pathway named reverse cholesterol transport by the ATP-binding cassette transporter A1 (ABCA1), ATP-binding cassette transporter G1 (ABCG1), and the scavenger receptor B1 (SR-B1) [91].

Recently, *SORT1* gene has also been identified to be expressed in macrophage. Evidence from previous clinical trials has strongly suggested a vital regulatory effect of sortilin in modulating the process of atherosclerosis through inducing foam cells formation, which reveals a relationship between sortilin and lipid metabolism within macrophage. For instance, an independent study already demonstrated the function of sortilin in modulating LDL uptake and foam cell formation. Besides, the authors also demonstrated that an *Apobec1* (-/-); *hAPOB* transgenic mice with *SORT1*-deficiency present no effect on regulating serum LDL-C levels, whereas with dramatically reduced atherosclerotic plaque areas within aortic root. Similarly, the *SORT1/LDLR*-DKO mice displayed reduced atherosclerotic plaque areas without any alteration in circulating levels of LDL-C, revealing that the recruitment of macrophage might not be the only mechanism whereby sortilin regulates the process of atherosclerosis [44]. As a consequence, the most possible mechanism might be that over-expressed *SORT1* gene within macrophage could affect the LDL-C endocytosis. Notably, the author also observed that macrophage with *SORT1*-deficiency embraced protective function which inhibited the process of atherosclerosis through suppressed LDL-C uptake and foam cells formation within macrophage [44], indicating a new mechanism of sortilin in regulating the intercellular lipid metabolism aside from the traditional endocytosis of LDL-C in macrophages, such as via the LDLR or via the macropinocytosis.

**FIGURE 3:**
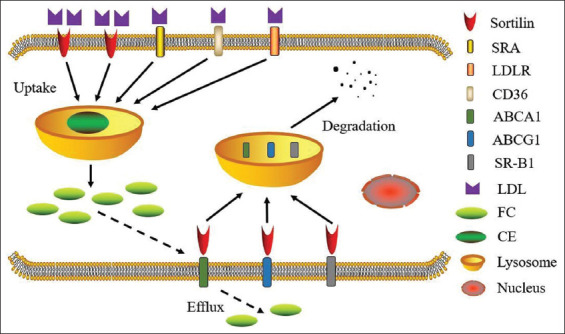
Mechanisms whereby sortilin influences lipid metabolism in macrophage. Sortilin could significantly modulate LDL uptake and foam cell formation within macrophage, further affecting the intra-cellular lipid metabolism. SRA: scavenger receptor A; LDLR: low-density lipoprotein receptor; CD36: cluster of differentiation 36; ABCA1: ATP-binding cassette transporter A1; ABCG1: ATP-binding cassette transporter G1; SR-B1: scavenger receptor B1; FC: free cholesterol; CE: cholesteryl ester.

The potential mechanisms whereby sortilin modulates the impaired cholesterol efflux from macrophage has been given substantial attention during the past several decades. According to the published reports, one of the main viewpoints is that the process of endocytosis of LDL particle induced by sortilin within macrophage might depend on serum concentrations of LDL-C. Under the condition of dyslipidemia, the exposure of macrophage to circulating LDL-C results in increased expression of *SORT1* gene and sortilin protein within macrophage. Subsequently, this process may induce an increased LDL-C endocytosis by sortilin [92]. Otherwise, the other viewpoint might be that over-expression of *SORT1* gene in macrophage up-regulates the content of sortilin protein, which could act as an intracellular sorting receptor and convey the lipid efflux transporters, such as ABCA1, ABCG1, and SR-B1, into the lysosome for subsequent degradation. In addition, this step leads to a significant reduction of lipid efflux mediated by macrophage [93]. Nonetheless, the precise mechanisms whereby sortilin affect intracellular lipid metabolism within macrophage need further investigation (Figure 3).

Conclusively, the underlying mechanisms whereby sortilin regulates serum or intra-cellular lipid metabolism within macrophage have been relatively elucidated. The sortilin level in macrophage has a close relationship with intercellular lipid metabolism and the inhibition of cholesterol efflux. Thereby, with excessive lipid storage within macrophage, sortilin facilitates the formation process of macrophage-derived foam cells and consequently promotes the pathological progression of atherosclerosis and its related coronary artery diseases.

## ROLE OF SORTILIN IN MODULATING SERUM GLUCOSE METABOLISM

In addition to the significant functions in influencing the serum or intra-cellular lipid catabolism, various glucose-regulatory effects have been also ascribed to sortilin. According to the emerging technological breakthroughs, several underlying potential mechanisms by which sortilin influence the serum glucose metabolism is being well-elucidated. As extensively shown in the previous studies, sortilin and GLUT4, as the most important transporter for glucose metabolism, are co-expressed within the adipocytes and myotubes, which have been demonstrated to be necessary for glucose accumulation [94]. Concerning this notion, fine-tuning of the expression contents of sortilin and myotubes are considered to be essential to maintain the insulin-induced intracellular transport of glucose. For instance, several research studies have shown that the formation of GLUT4-accumulated vesicles within the adipocyte and skeletal muscle cells was closely associated with the gene expression content of *SORT1* [82,95]. Moreover, the insulin-mediated translocation of GLUT4 has also been shown to be strongly correlated with the gene expression contents of *SORT1* within the adipocyte [81]. Concerning this notion, it has been speculated that the disrupted transport of glucose which was promoted by the pathogenic progression of insulin resistance under the condition of cardiometabolic syndrome, was associated with the alteration of the expression contents of sortilin [96].

On the other hand, the gene expression content of *SORT1* has also been shown to be reduced under multiple pathological statuses, such as diabetes mellitus, hypertension, and obesity. This significant inhibition has been further confirmed to be mediated by several pro-inflammatory cytokines, including TNF-α. Intriguingly, Kaddai et al. have postulated a potential relationship between the chronic inflammatory response, the serum sortilin concentrations, and the risk of diabetes mellitus [96]. Actually, the serum expression levels of sortilin are significantly altered in the mouse models with insulin resistance promoted by TNF-α or the intervention of dexamethasone. Consistent with this result, in presence of TNF-α, the expression of *SORT1* gene was confirmed to be significantly reduced and could be closely correlated with insulin resistance. Nonetheless, on the contrary, the dexamethasone-mediated insulin resistance is shown to be not accompanied by the reduced gene expression contents of *SORT1* [97,98].

With in-depth investigations, the expression of *SORT1* gene was confirmed to be significantly reduced within adipocytes isolated from the obese mice, especially after the PI3K/AKT signaling pathway is inhibited, indicating that the insulin-dependent signal pathway could modulate the expression of sortilin [99]. In addition, it has been shown that both TNF-α and dexamethasone could significantly inhibit the biological activity of insulin receptor tyrosine kinase, which subsequently facilitated the insulin resistance without influencing the translocation of GLUT4 [100,101]. Taken together, the results mentioned above indicate that the reduced expression of *SORT1* gene could be identified as one of the most important biomarkers which results in insulin resistance

## CONCLUSION

As described above, there is considerable evidence linking the significant variability at the *SORT1* gene locus with the increased risk of dyslipidemia and its related cardio-metabolic syndrome. Though the potential mechanism is needed to be further elucidated, it is reasonable to make a speculation to conclude that the relationships between higher serum levels of LDL-C is closely correlated with the *SORT1* SNPs in patients worldwide. On the other hand, almost all the published results provided an unequivocal function of *SORT1* SNPs in regulating lipid metabolism, shedding light on that the effect of 1p13.3 gene locus could be identified as a novel biomarker representing the risk of dyslipidemia and its related cardiometabolic syndrome, such as overweight/obesity, atherosclerotic coronary diseases, and diabetes mellitus. Aside from the regulatory role of *SORT1* gene SNPs, the expression of sortilin in hepatocyte, adipocyte, and macrophage indicates that sortilin could be considered as an essential factor in controlling intracellular lipid catabolism and resultantly influences the pathological process of cardiometabolic syndrome.

Although multiple breakthroughs have been provided to deeply elucidate the relationship between the alterations of circulating sortilin and the risk of dyslipidemia due to the technological advances, some reports verified the contradictory effect of over-expressed *SORT1* gene on modulating the circulating level of LDL-C. To date, the accurate biological function of sortilin seem to be clarified, it is still worth noting that the effect of sortilin in regulating circulating and intercellular lipid catabolism is controversial. We need large-scale and more comprehensive study to further elucidate the modulatory function of sortilin within hepatocyte, adipocyte, and macrophage.
